# Urothelial Bladder Carcinoma Incidentally Coexisting with an Ileovesical Fistula Caused by Perforation of Meckel’s Diverticulum: A Case Report

**DOI:** 10.3390/reports9020119

**Published:** 2026-04-13

**Authors:** Atsushi Yamamoto, Shohei Kawaguchi, Satoko Urata, Sotaro Miwa

**Affiliations:** Department of Urology, National Hospital Organization (NHO) Kanazawa Medical Center, Kanazawa 920-8650, Japan

**Keywords:** ileovesical fistula, Meckel’s diverticulum, bladder cancer

## Abstract

**Background and Clinical Significance**: Enterovesical fistulas are uncommon and are most often caused by inflammatory conditions. Cases caused by Meckel’s diverticulum are exceptionally rare, with only a few cases reported in the literature. To our knowledge, no previous reports have described an ileovesical fistula due to Meckel’s diverticulum coexisting with urothelial carcinoma. **Case Presentation**: A 74-year-old man was evaluated after presenting with fever and diagnosed with an ileovesical fistula. Since cystoscopy findings could not completely exclude bladder cancer, transurethral resection of the bladder tumor (TUR-Bt) was performed 1 month after the infection subsided. Histopathological examination confirmed the diagnosis of bladder cancer. Partial small intestine resection was performed 1 week after TUR-Bt to treat the ileovesical fistula. Histopathological examination of the resected specimen revealed perforation due to Meckel’s diverticulitis. TUR-Bt was repeated 3 months after the initial surgery, and no residual tumor was detected. At 6 months postoperatively, no recurrence of bladder cancer or fistula was observed. **Conclusions**: This case highlights an extremely rare coexistence of ileovesical fistula due to Meckel’s diverticulum and urothelial carcinoma. Given the potential for malignancy in or around fistulous lesions, careful diagnostic evaluation and appropriate management are essential.

## 1. Introduction and Clinical Significance

Enterovesical fistula is an uncommon condition, mainly caused by inflammation, but it can also result from neoplastic or traumatic factors [1]. Ileovesical fistulas represent about 16% of all enterovesical fistulas [2] and are uncommon among inflammatory types. Cases caused by Meckel’s diverticulum are exceptionally rare, with only a few cases reported in the literature [3]. Although several cases of enterovesical fistula associated with malignancy have been reported [4,5,6,7], no cases of ileovesical fistula caused by Meckel’s diverticulum complicated by urothelial carcinoma have been described so far. Herein, we present a rare case of ileovesical fistula due to Meckel’s diverticulitis concomitant with urothelial carcinoma, along with a review of the relevant literature.

## 2. Case Presentation

A 74-year-old man presented to a local urology clinic with swelling and pain in the left scrotum and was diagnosed with left epididymitis. He was a former smoker with a 53-year history of smoking 30 cigarettes per day. His medical history included benign prostatic hyperplasia, type 2 diabetes mellitus, cerebral infarction, hypertension, nephrosclerosis, arteriosclerosis obliterans, and hyperuricemia. His medication included tamsulosin, aspirin, clopidogrel, atorvastatin, omega-3 fatty acid ethyl esters, allopurinol, sacubitril/valsartan, esaxerenone, nifedipine, and sodium ferrous citrate. He had a history of appendectomy and endoscopic submucosal dissection for early gastric cancer. In addition, he had a known allergy to contrast media (iomeprol). Despite antibiotic therapy, left testicular pain persisted, and he subsequently developed a high-grade fever of ~39 °C. Despite no prior history, he reported fecaluria prior to admission. He was therefore transferred to our hospital on an emergency basis.

**Figure 1:**
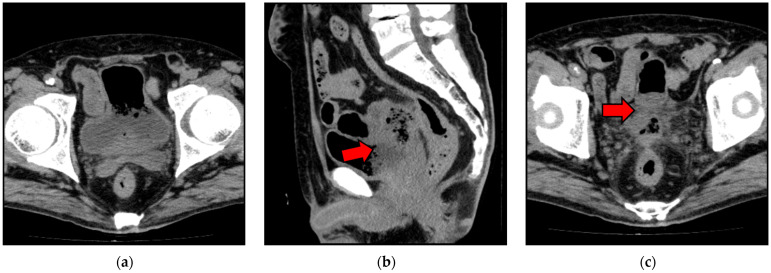
Residual debris-like shadows and intravesical gas accumulation in the urinary bladder (**a**). Although the fistula tract is not clearly delineated on CT, findings suggest a possible connection between the ileum and urinary bladder (**b**,**c**). Arrows indicate the suspected fistula region.

On admission, together with findings consistent with left epididymitis, non-contrast computed tomography (CT) revealed debris and gas accumulation within the urinary bladder; however, the fistula tract was not clearly delineated. These findings suggested an enterovesical fistula. Although the fistula tract was not clearly visualized on CT, the presence of intravesical gas and debris strongly supported the suspicion of an enterovesical fistula (Figure 1). Although meropenem was initiated as antimicrobial therapy, the patient developed sepsis and disseminated intravascular coagulation. Accordingly, recombinant thrombomodulin alfa was started on hospitalization day 1.

Subsequently, the systolic blood pressure decreased to 90 mmHg and, despite noradrenaline administration, the patient remained hemodynamically unstable. Blood cultures yielded Gram-negative rods, leading to a diagnosis of endotoxin shock. Therefore, endotoxin adsorption therapy and continuous hemodiafiltration were initiated. The patient’s hemodynamic status improved the following day, and noradrenaline was discontinued.

**Figure 2:**
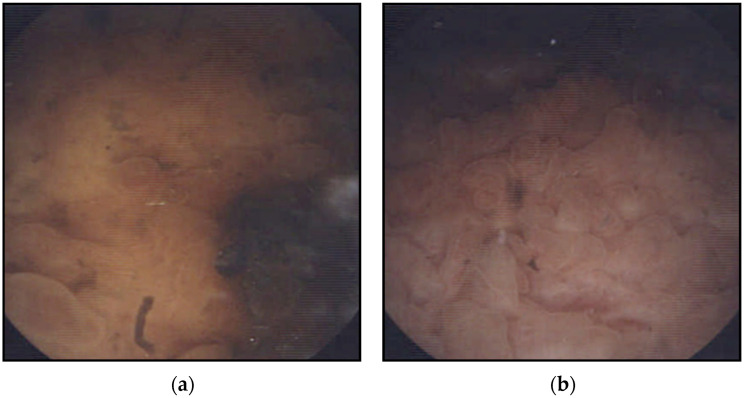
A perforation in the posterior wall of the urinary bladder, with leakage of intestinal contents from the same site. In addition, irregularity of the surrounding bladder mucosa (**a**,**b**).

**Figure 3:**
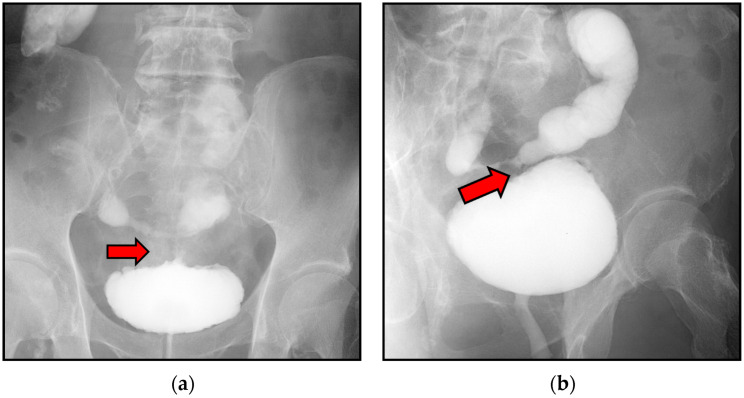
Contrast medium extravasation from the posterior wall of the urinary bladder into the ileum, clearly demonstrating the ileovesical fistula (**a**,**b**). Arrows indicate the fistula tract.

Contrast-enhanced CT showed enhancement of the fistula wall but no obvious tumorous lesions. No oral contrast was administered for CT. Similarly, plain magnetic resonance imaging (MRI) showed no tumorous lesions in either the urinary bladder or ileum. A lower gastrointestinal endoscopy revealed no tumorous lesions or fistulas in the sigmoid colon or rectum, but cystoscopy demonstrated a perforation in the posterior wall of the urinary bladder, where fecal discharge was observed. In addition, an area of irregular mucosa was noted around the fistula, and bladder cancer could not be completely excluded (Figure 2). The urinary cytology from bladder washing was negative, but cystography demonstrated contrast medium leakage from the posterior wall of the urinary bladder into the ileum, confirming the presence of an ileovesical fistula. Cystography played a key role in establishing a definitive diagnosis (Figure 3). Additional imaging modalities, such as magnetic resonance (MR) enterography or dedicated small bowel imaging, were not performed as contrast-enhanced CT and cystography were considered sufficient for diagnosis.

**Figure 4:**
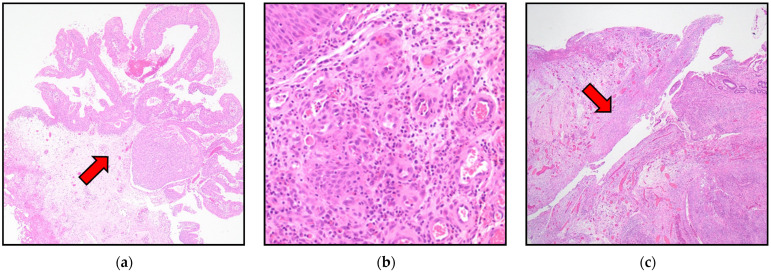
Papillary urothelial carcinoma without stromal invasion (pTa) (hematoxylin and eosin(H&E) staining, ×40) (**a**). Granulation tissue formation and dense fibrosis in the submucosa adjacent to the fistula, consistent with chronic inflammatory changes (H&E staining, ×200) (**b**). Resected Meckel’s diverticulum showing perforation (H&E staining, ×20) (**c**). Arrows indicate the relevant pathological findings in each panel.

On hospital day 31, TUR-Bt was performed and histopathological examination revealed noninvasive papillary urothelial carcinoma (G1–2, pTa). In addition, granulation tissue formation and dense fibrosis in the submucosa adjacent to the fistula, together with urothelial hyperplasia and squamous metaplasia, suggested chronic inflammatory changes (Figure 4). On hospital day 37, partial resection of the small intestine was performed to treat the ileovesical fistula. To prevent intraperitoneal dissemination of the bladder cancer, the urinary bladder was not opened; only the intestinal segment was resected. Histopathological examination of the resected specimen revealed a perforated Meckel’s diverticulum with ectopic gastric tissue and surrounding chronic inflammatory changes, without evidence of dysplasia, malignancy, or findings suggestive of Crohn’s disease, suggesting that the ileovesical fistula was caused by perforation due to Meckel’s diverticulitis (Figure 4). The patient was discharged on hospital day 52. A voiding cystography performed 28 days after intestinal surgery confirmed the absence of contrast leakage, and the urethral catheter was subsequently removed. Thereafter, urinary function was satisfactory. A second TUR-Bt 3 months after the initial one and histopathological examination revealed no residual tumor. To exclude malignancy, TUR-Bt was performed before intestinal surgery, as cystoscopic findings revealed an irregular mucosal lesion suspicious for bladder cancer. Establishing a definitive diagnosis was considered essential for determining the optimal surgical strategy. Intestinal surgery was postponed until the patient’s overall condition improved, as initial presentation included septic shock and disseminated intravascular coagulation necessitating intensive care management. A second TUR-Bt was performed to confirm complete resection, given the initial pathological findings of non-muscle-invasive bladder cancer and the potential risk of residual tumor. Intravesical therapy, including bacillus Calmette–Guérin (BCG) or intravesical chemotherapy, was not administered as no residual tumor was identified on repeat TUR-Bt, and no additional treatment was considered necessary.

No recurrence of bladder cancer or the fistula was observed at 6 months postoperatively. A timeline of the clinical course is presented in Table 1.

## 3. Discussion

Despite numerous reports of enterovesical fistulas and intestinal diverticula, enterovesical fistulas caused by Meckel’s diverticulum have been only sporadically reported [8]. Clinical manifestations of enterovesical fistula include pneumaturia, fecaluria, urinary frequency, fever, urinary tract infection, abdominal pain, hematuria, and passage of urine through the rectum [9]. In the present case, an enterovesical fistula was suspected based on the presence of fever and fecaluria, and CT confirmed fistula formation between the ileum and urinary bladder. Contrast-enhanced CT has been reported as useful for diagnosing enterovesical fistula [10]. In this case, while CT results were suggestive, cystography was essential for confirming the diagnosis.

The origin of enterovesical fistulas is generally classified as inflammatory, neoplastic, or traumatic, with inflammatory conditions being the most common [1]. Among inflammatory causes, diverticulitis is the most frequent, accounting for ~65–79% of cases, which are typically colovesical fistulas. The next most common causes are malignancy (10–20%) and Crohn’s disease (5–7%) [9]. In the present case, dense adhesions between the small intestine and the peritoneum were observed. Histopathological examination revealed tissue in the ileal mucosa resembling gastric pyloric glands. These findings suggested that the ileovesical fistula developed secondary to inflammation of the Meckel’s diverticulum.

Regarding treatment, there are reports describing successful outcomes after partial resection of both the small intestine and bladder, including the fistulous tract [11]. Conversely, some studies have indicated that no additional bladder procedure is required if intraoperative cystography shows no leakage of contrast material from the fistula [12]. In cases of ileovesical fistulas caused by Meckel’s diverticulum, the involved bowel was divided using a stapling device, followed by resection of the diverticulum and a segment of the small intestine [8]. In reports of advanced bladder cancer (urothelial carcinoma or squamous cell carcinoma) forming fistulas with the small intestine [4,5], bladder cancer directly invaded the small intestine and resulted in fistula formation. In addition, cases of ileovesical fistula caused by primary intestinal adenocarcinoma [7] and cases where small intestinal cancer developed from an ileovesical fistula associated with Crohn’s disease have been reported [6]. However, despite six reports of ileovesical fistula caused by perforation of a Meckel’s diverticulum [3], to the best of our knowledge, this is the first report of urothelial carcinoma in the perilesional area of an ileovesical fistula secondary to Meckel’s diverticulum. Regarding the causal relationship between fistulas and small intestinal adenocarcinoma, fistulas may represent ulcerative lesions with chronic mucosal regeneration and repair. During this process, a regenerated epithelium with high-grade dysplasia may develop, which could act as a potential precancerous lesion [6,13].

In the present case, the diagnosis of urothelial carcinoma of the bladder resulted from a TUR-Bt performed before partial resection of the small intestine. Thereafter, the possibility of incomplete tumor resection could not be completely excluded. If partial cystectomy with bladder opening had been performed, there would be concern about intraperitoneal tumor cell spread, so the surgical approach required careful consideration.

On the other hand, based on imaging findings and histopathological results, the bladder cancer was superficial (pTa) and its direct involvement in fistula formation was considered unlikely.

There was no radiological or intraoperative evidence of tumor invasion into the surrounding tissues.

Partial or radical cystectomy was considered but rejected as too invasive, since the tumor was superficial and there was no evidence of tumor involvement in the fistula. In addition, bladder opening was avoided to minimize the risk of intraperitoneal tumor dissemination. These treatment strategies were comprehensively discussed with the patient and his family; however, the patient declined to undergo a highly invasive surgical intervention.

Intraoperative cystography revealed no leakage of contrast material. Therefore, no partial cystectomy was performed; instead, the small intestine was mobilized and the bladder wall was approximated and sutured.

When advanced bladder cancer is involved in formation of an enterovesical fistula, most cases are invasive carcinomas with muscular invasion [4,5]. In the present case, CT and MRI showed no evidence of bladder wall perforation or obvious tumor invasion, and histopathological examination demonstrated a tumor invasion depth of pTa. The relationship between the bladder tumor and fistula formation remains unclear. Given the pathological findings and the lack of evidence of tumor involvement in the fistula tract, the urothelial carcinoma was considered an incidental finding. Regarding fistula-associated malignancies, multiple cases have been documented as adenocarcinoma [6,7,13], while reports identifying a relationship with urothelial carcinoma, as observed in the present case, remain limited. This suggests a distinct pathological background for urothelial carcinoma in this context. Therefore, depending on the tumor progression pattern and histological type, treatment strategies that prioritize oncological radicality, including partial or radical cystectomy, may be considered even in situations with a possible risk of tumor dissemination associated with a fistula.

Previously reported cases of ileovesical fistula caused by Meckel’s diverticulum are summarized in Table 2. Notably, some were associated with Crohn’s disease or foreign bodies, and no previous reports have described concomitant urothelial carcinoma.

## 4. Conclusions

This case illustrates an extremely rare condition in which urothelial carcinoma incidentally coexisted with an ileovesical fistula caused by perforation of Meckel’s diverticulum. Furthermore, since cases with fistula formation may be complicated by an associated malignancy within or around the fistula, careful evaluation is required for both diagnosis and treatment.

## Figures and Tables

**Table 1:** Timeline of clinical course.

Time	Clinical Event	Details
Day—12	Symptom onset	Left scrotal pain and swelling; epididymitis diagnosis at a local clinic
Day 0	Hospital admission	High fever (~39 °C); CT revealed intravesical gas and debris, suggesting an enterovesical fistula
Day 1	Clinical deterioration	Septic shock and disseminated intravascular coagulation; intensive care initiated, including recombinant thrombomodulin
Day 2–4	Intensive treatment	Endotoxin adsorption therapy and continuous hemodiafiltration; hemodynamic stabilization
Day 31	TUR-Bt	Performed due to suspicion of bladder cancer; histopathology showed non-muscle-invasive urothelial carcinoma (pTa, G1–2)
Day 37	Intestinal surgery	Partial small intestine resection for ileovesical fistula; Meckel’s diverticulitis confirmed histologically
Postoperative Day 28	Follow-up cystography	No contrast leakage; urethral catheter removed
3 months after TUR-Bt	Second TUR-Bt	No residual tumor detected
6 months after surgery	Follow-up	No recurrence of bladder cancer or fistula

**Table 2:** Reported cases of ileovesical fistula associated with Meckel’s diverticulum. “Not described” indicates that the information was not explicitly reported in the original articles.

Author (Year)	Age/Sex	Etiology	Symptoms	Treatment
Aubert [14] (1971)	Not described	Meckel’s diverticulum	Not described	Not described
Dearden [15] (1983)	81/F	Meckel’s diverticulum	Abdominal pain, dysuria, frequency, nausea/vomiting	Resection with bladder repair
Mackenzie [16] (1989)	30/F	Meckel’s diverticulum	Not described	Diverticulectomy with resection of bladder cuff
Petros [17] (1990)	22/M	Crohn’s ileitis with Meckel’s diverticulum	Fever, dysuria	Diverticulectomy and terminal ileal resection
Hudson [18] (1992)	23/M	Meckel’s diverticulum	Abdominal pain, diarrhea	Diverticulectomy
Graziotti [19] (2002)	40/M	Foreign body in Meckel’s diverticulum	Recurrent dysuria, fever	Ileal resection with partial bladder resection and foreign body removal
Present case	74/M	Meckel’s diverticulitis	Fecaluria, fever	Partial small bowel resection

## Data Availability

The original data presented in this study are available on reasonable request from the corresponding author. The data are not publicly available due to privacy concerns.

## Abbreviations

The following abbreviations are used in this manuscript:

TUR-Bt	Transurethral resection of the bladder tumor
CT	Computed tomography
MRI	Magnetic resonance imaging
H&E	Hematoxylin and eosin

## References

[1] Sellers W., Fiorelli R. (2015). Enterovesical fistula secondary to squamous cell carcinoma of the bladder. Urol. Case Rep..

[2] Céspedes Rodríguez H.A., Tello Duanes D.A. (2022). Colovesical fistula, as a manifestation of cancer of the left colon: Case presentation. MedCrave Online J. Surg..

[3] Bouassida M., Mighri M.M., Trigui K., Chtourou M.F., Sassi S., Feidi B., Chebbi F., Bouzaidi K., Touinsi H., Sassi S. (2013). Meckel’s diverticulum: An exceptional cause of vesicoenteric fistula: Case report and literature review. Pan Afr. Med. J..

[4] Ng Z.Q., Low W.K.W., Sathiyananthan, Subramanian P., Stein J. (2017). Radical cystectomy and en-bloc resection of enterovesical fistula from bladder cancer. World J. Clin. Urol..

[5] Kang Y.J., Park D.J., Kim S., Kim S.W., Lee K.S., Choi N.G., Kim K.H. (2014). Vesicoenteric fistula due to bladder squamous cell carcinoma. Korean J. Urol..

[6] Tada K., Ueda Y., Kusano T., Etoh T., Inomata M., Kitano S. (2012). A case of adenocarcinoma in a Crohn’s disease enterovesical fistula. J. Jpn. Surg. Assoc..

[7] da Silva Corrêa R., Macena Salviano F.A., Revorêdo Antunes de Melo L.F., Dantas Junior J.M., Guedes Pereira Brandão I.R., Pinheiro T.B. (2018). Enterovesical fistula caused by ileal primary adenocarcinoma. J. Coloproctol..

[8] Hakoda H., Mishima H., Habu T., Murai S., Maeno R., Yokomizo Y., Inagaki Y., Maruyama T., Matsui Y., Sako A. (2018). Laparoscopic treatment of a vesicointestinal fistula due to a Meckel’s diverticulum: A case report and review of the literature. Clin. J. Gastroenterol..

[9] Gill H.S. (2016). Diagnosis and surgical management of uroenteric fistula. Surg. Clin. N. Am..

[10] Golabek T., Szymanska A., Szopinski T., Bukowczan J., Furmanek M., Powroznik J., Chlosta P. (2013). Enterovesical fistulae: Aetiology, imaging, and management. Gastroenterol. Res. Pract..

[11] Han S.-R., Kim H.-J., Yoo R.N., Shin S.H., Kim G., Cho H.M., Lee S.-J., Lee H.-I. (2021). Enterovesical fistula from Meckel diverticulum. Ann. Coloproctol..

[12] Dziki Ł., Włodarczyk M., Sobolewska-Włodarczyk A., Mik M., Trzciński R., Hill A.G., Dziki A. (2019). Is suturing of the bladder defect in benign Enterovesical fistula necessary?. BMC Surg..

[13] Laurent S., Barbeaux A., Detroz B., Detry O., Louis E., Belaiche J., Meurisse M. (2005). Development of adenocarcinoma in chronic fistula in Crohn’s disease. Acta Gastroenterol. Belg..

[14] Aubert J. (1971). Ileovesical fistula due to Meckel’s diverticulum. A case; literature review. J. Urol. Nephrol..

[15] Dearden C., Humphreys W.G. (1983). Meckel’s diverticulum: A vesico diverticular fistula. Ulster Med. J..

[16] Mackenzie T.M., Kisner C.D., Murray J. (1989). Vesicoileal fistula via Meckel diverticulum. Urology.

[17] Petros J., Argy O. (1990). Enterovesical fistula from Meckel’s diverticulum in a patient with Crohn’s ileitis. Dig. Dis. Sci..

[18] Hudson H.M., Millham F.M., Dennis R. (1992). Vesico-diverticular fistula: A rare complication of Meckel’s diverticulum. Am. Surg..

[19] Graziotti P., Maffezzini M., Candiano G., Maugeri O. (2002). Vesicoenteric fistula created by ingested foreign body in Meckel’s diverticulum. J. Urol..

